# Manifestations and Outcomes of Patients with Parkinson's Disease and Serious Infection in the Emergency Department

**DOI:** 10.1155/2018/6014896

**Published:** 2018-10-17

**Authors:** Chih-Min Su, Chia-Te Kung, Fu-Cheng Chen, Hsien-Hung Cheng, Sheng-Yuan Hsiao, Yun-Ru Lai, Chin-Cheng Huang, Nai-Wen Tsai, Cheng-Hsien Lu

**Affiliations:** ^1^Department of Emergency Medicine, Kaohsiung Chang Gung Memorial Hospital, Chang Gung University College of Medicine, Kaohsiung, Taiwan; ^2^Chung Shan Medical University, School of Medicine, Taiwan; ^3^Department of Biological Science, National Sun Yat-Sen University, Kaohsiung, Taiwan; ^4^Department of Neurology, Kaohsiung Chang Gung Memorial Hospital, Chang Gung University College of Medicine, Kaohsiung, Taiwan; ^5^Center for Shockwave Medicine and Tissue Engineering, Kaohsiung Chang Gung Memorial Hospital, Chang Gung University College of Medicine, Kaohsiung, Taiwan; ^6^Department of Neurology, Xiamen Chang Gung Memorial Hospital, Xiamen, Fujian, China

## Abstract

**Background:**

Several comorbidities contribute to an increased risk of infections in Parkinson's disease (PD) as the disease progresses. However, few studies have examined the correlation between sepsis and PD.

**Aim:**

The aim of this study is to disclose the presentation and outcome of serious infection in patients with PD in the emergency department.

**Methods:**

This retrospective cohort study enrolled patients with PD who had serious infection and were admitted to the emergency department between January 2007 and December 2013. For clinical comparison, we compared the clinical features, laboratory data, and outcomes with those of age- and sex-matched patients who had serious infection but not PD.

**Results:**

There were a total of 1,200 episodes of infected PD patients and 2,400 age- and sex-matched infected patients without PD as disease controls. PD patients had fewer comorbidities and lower severity of infectious disease but longer hospital stays than control group patients. The incidences of respiratory tract and urinary tract infections were higher in PD patients. The levels of inflammatory and organ dysfunction biomarkers in PD were lower and compatible with the severity of infectious disease. A total of 86 (7.2%) infected PD patients died during the 28-day admission compared to 339 (14.1%) in non-PD patients. Serum C-reactive protein, bandemia, and lactate could be used to predict mortality in infected PD patients.

**Conclusions:**

In infected patients with PD, respiratory and urinary tract infections were the two most common infectious sources. Empiric therapy based on experience could treat both respiratory and urinary tract infections. Early diagnosis and treatment are essential for survival.

## 1. Introduction

Parkinson's disease (PD), the second most common neurodegenerative disease after Alzheimer's disease, includes both motor and nonmotor symptoms [1]. The features of the motor symptoms include resting tremor, rigidity, and bradykinesia [2]. As the disease progresses, hypokinesia of face muscles and a monotone change of the voice become involved and instability, rigidity worsen, and postural instability. Finally, the disease progresses to easy choking and bedridden status. Therefore, infection is a common problem in patients with advanced PD. Several nationwide studies have demonstrated that respiratory tract and urinary tract infections are the leading cause of acute emergency admission in PD patients [3–5]. Since the number of PD patients is expected to double by the year 2030, how to manage infected PD patients in the emergency department (ED) is becoming an important issue [5].

Several comorbidities (e.g., easy choking and bedridden status) contribute to the increased risk of infections in PD, but nonmotor symptomatology (e.g., autonomic dysfunction, dementia, and depression) and medications (e.g., dopaminergic or anticholinergic medications, which could cause orthostatic hypotension and mental change in elderly patients, which may mask the systemic inflammatory response) can interfere the diagnosis [6, 7].

Given that sepsis can be fatal, determining the clinical manifestations associated with serious infection in patients with PD could prevent delayed diagnosis. Here we aimed to analyze clinical features including infection site, laboratory data, blood culture results, hospital stay, and eventual outcome associated with serious infection. Through this study, we surveyed all infected PD patients who were admitted to the ED and compared them with age- and sex-matched infected patients to improve the therapeutic strategy.

## 2. Materials and Methods

### 2.1. Study Design

We conducted this single-center retrospective observational study on PD patients admitted to the ED with suspected serious infection, at least one set of blood culture tests, and intravenous antibiotic therapy. The data were retrieved from computerized medical records between 1 January 2007 and 31 December 2013 from Kaohsiung Chang Cheng Memorial Hospital, a 2692-bed acute-care teaching hospital, the largest medical center in Southern Taiwan providing both primary and tertiary referral care. The institutional review board of Chang Cheng Memorial Hospital approved this study and waived the requirement for informed consent.

### 2.2. Study Setting and Population

**Figure 1:**
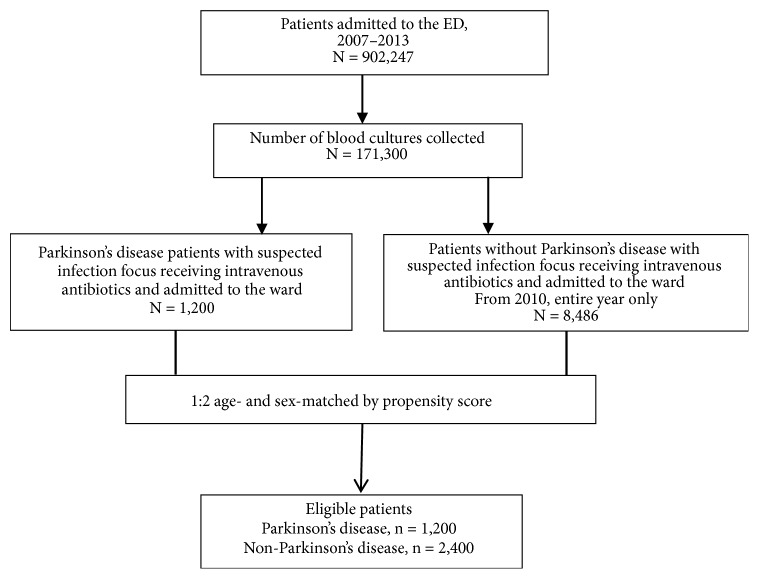
Study flow chart.

All the patients from whom blood culture samples were collected in the ED were screened in a computer database as shown in Figure 1. Patients who were admitted and received intravenous antibiotics were further evaluated. All patients with PD were enrolled in the study. A ratio of 1:2 age- and sex-matched control group patients were selected by propensity score matching from the 2010 whole year patients with the same criteria. For all enrolled patients, the following data were collected retrospectively: demographic characteristics, preexisting major comorbidities, initial vital signs and laboratory tests results, major infection source, and microorganisms isolated from the blood cultures.

Blood culture samples with potential contaminating pathogens (e.g., coagulase-negative* Staphylococcus*,* Propionibacterium acnes*,* Micrococcus*,* Corynebacterium spp*., and* Peptostreptococcus*) were considered contaminations and were regarded as no bacteremia [8]. Laboratory data represented the result of the first test done in the ED. Septic shock is defined as sepsis-induced hypotension persisting despite adequate fluid resuscitation and inotropic agent use [9]. The major outcome was defined as 28-day in-hospital mortality.

### 2.3. Clinical Definitions

Since all patients were admitted for treatment, discharge diagnosis records were available for them in the database. The underlying disease and major infection focus were obtained from ICD-9 coding. PD was defined by specific ICD-9 coding (332.0) plus the use of Parkinsonism medication. Other comorbid underlying diseases defined by ICD-9 coding included liver cirrhosis (571.2, 571.5, 571.6), hypertension (400.9–405.1), diabetes mellitus (250.00–250.99), congestive heart failure (428.0–428.9), renal insufficiency (582.00–589.99), malignancy (140.00–199.99), hematological disease (200.00–208.99), autoimmune disease (710.0–714.9), and stroke (430.00–438.99). Major infection focus including respiratory tract infection (481.0–486.9), urinary tract infection (590.00–590.99, 601.0–601.9), biliary tract infection (576.1, 574.00–574.19, 574.30–574.49, 574.60–574.89), intra-abdominal infection (562.11, 567.0–567.9), soft tissue infection (680.0–686.9, 728.86), and meningitis (320.00–320.99, 324.0–324.9) were defined by ICD-9 coding accordingly, while others not belonging to the above six categories were considered unknown.

### 2.4. Statistical Analysis

The statistical analyses were performed using the Statistical Package for the Social Sciences for Windows version 20.0 (SPSS, Chicago, IL, USA). Continuous variables are expressed as mean ± SD and were compared using Student's t-test. Categorical variables, expressed as numbers and percentages, were compared using the *χ*^2^ test or Fisher's exact test. All significant variables (P values < 0.05) on univariate analysis were incorporated into a hierarchical logistic regression model. We also created Cox proportional hazards model to estimate covariate-adjusted survival. P values < 0.05 were considered statistically significant.

## 3. Results

### 3.1. Demographic Data of PD Patients with Serious Infection

A total of 1,200 episodes of infection events occurred in the PD patients (n = 782) during this 7-year period. Of them, 696 (89%) patients had only one episode of infection, while only 12 patients (1.5%) had >6 infection events.  Table 1 shows that PD patients had lower percentages of liver cirrhosis (2.8% vs. 7.8%, p<0.001), congestive heart failure (5.9% vs. 11.3%, p<0.001), malignancy (9.5% vs. 22.8%, p<0.001), hematological disease (0.8% vs. 2.4%, p<0.001), and autoimmune disease (0.6% vs. 1.4%, p=0.041) than those without PD. PD patients were at higher risk of developing respiratory tract infection (49.5% vs. 43.6%, p=0.001) and urinary tract infection (48.6% vs. 23.3%, p<0.001) but were at lower risk of developing biliary tract infection (2.4% vs. 7.1%, p<0.001) and intra-abdominal infection (1.4% vs. 3.6%, p<0.001). The indications for the severity of infection including intensive care unit (ICU) admission, septic shock, and respiratory failure all showed that PD patients were less severe than their age- and sex-matched patients. The overall in-hospital mortality rate of infected PD patients was 7.2%, which was significantly lower than the 14.1% of non-PD patients (p<0.001). We created a multivariate regression model to predict mortality among all enrolled patients and found that PD patients had a significantly lower odds ratio (OR, 0.616; 95% confidence interval [CI], 0.474–0.802). Although it seemed that admitted PD patients had less severe sepsis, they had longer hospital stays than overall patients (17.8 days vs. 15.4 days, p<0.001) or survival-only patients (18.1 days vs. 15.5 days, p<0.001).  Table 2 shows that the PD patients had lower levels of inflammatory and organ dysfunction biomarkers, which also inferred lower infectious disease severity. There was no difference in the percentage of bacteremia or the distribution of bacterial species between the two groups (Table 3). The leading three causative bacteria in the septic PD patients were* Escherichia coli*,* Streptococcus spp.*,* Klebsiella pneumoniae,* and in non-PD patients were* Escherichia coli, Klebsiella pneumoniae, *and* Staphylococcus aureus*.

### 3.2. Risk Stratification of PD Patients with Serious Infection

Table 4 shows that patients with renal insufficiency and cancer were at a higher risk of mortality. Elderly patients with lower blood pressure at the ED were also prone to bad outcomes. Respiratory tract infection carried a higher risk of mortality than other infection sites; in contrast, urinary tract infection carried the lowest risk of mortality. After we adjusted for age and sex for all significant comorbidity and infection focus factors, we found that age (OR, 1.039; 95% CI, 1.002–1.077), female sex (OR, 2.413; 95% CI, 1.413–4.121), respiratory tract infection (OR, 2.451; 95% CI, 1.365–4.403), cancer (OR, 2.804; 95% CI, 1.468–5.536), and renal insufficiency (OR, 3.793; 95% CI, 2.028–7.097) were significant risk factors of sepsis-related mortality, whereas urinary tract infection (OR, 0.340; 95% CI, 0.185–0.624) carried a higher chance of survival.

### 3.3. Inflammatory Biomarkers in PD Patients

The presentation of infectious biomarkers among PD patients with or without mortality is shown in Table 5. Serum C-reactive protein (CRP), bandemia, and lactate levels were good indications for prediction of 28-day in-hospital mortality of infected PD patients. On receiver operating characteristic curve analysis, CRP, bandemia, and lactate had areas under the curve (AUC) of 0.648 (95% CI, 0.575–0.721), 0.656 (95% CI, 0.588–0.724), and 0.653 (95% CI, 0.556–0.750), respectively, with different enrolled case numbers.

### 3.4. Survival Analysis of Infected PD Patients

**Figure 2:**
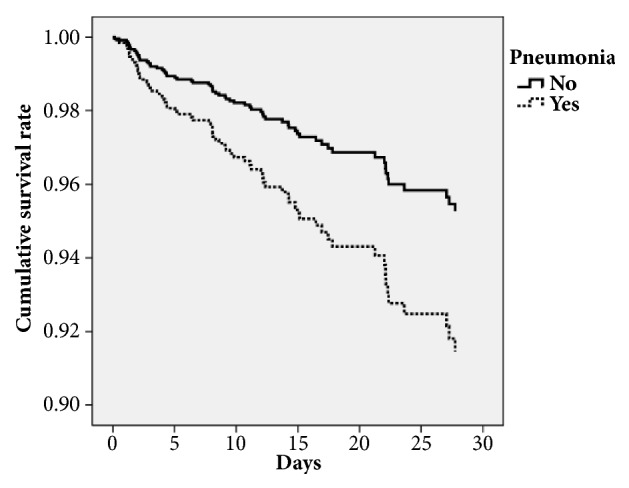
Survival curve of Cox regression model categorized by pneumonia status.

**Figure 3:**
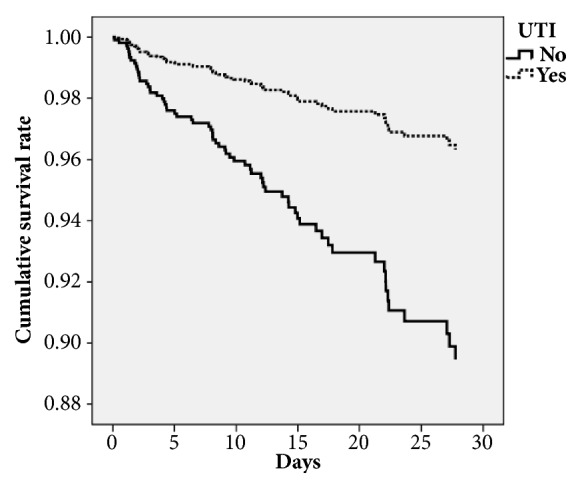
Survival curve of Cox regression model categorized by urinary tract infection status.

We also performed a survival analysis of the infected PD patients. We entered age, sex, pneumonia, urinary tract infection, cancer, and renal insufficiency into the Cox regression model. It revealed that pneumonia (OR, 1.842; 95% CI, 1.057–3.211), urinary tract infection (OR, 0.337; 95% CI, 0.188–0.604), age (OR, 1.037; 95% CI, 1.001–1.073), female (OR, 2.262; 95% CI, 1.369–3.737), renal insufficiency (OR, 2.850; 95% CI, 1.634–4.972), and cancer (OR, 2.063; 95% CI, 1.154–3.688) had different hazard ratios in the model. Figures 2 and 3 show the survival curve by infection site categories including pneumonia and urinary tract infection.

## 4. Discussion

To our knowledge, this is the first study to compare the clinical features and therapeutic outcomes of infected patients with or without PD. In the present study, we produced two major findings. First, the PD group was more likely to have respiratory tract and urinary tract infections than the age- and sex-matched patients. Second, although the PD group had a more benign course and lower fatality rate, they had longer mean hospitalization than the non-PD group.

The respiratory tract and the urinary tract were the two most common infection sites among PD patients in this and other studies [5, 10]. Our study revealed that pneumonia is the leading cause of sepsis-related mortality in PD patients; in contrast, urinary tract infections had the highest survival rates. PD patients were at risk of pulmonary complications as a consequence of changes in ventilation parameters and dysphagia [11]. One study showed a link between treated dental caries and a decreased risk of pneumonia among PD patients, which suggests that aspiration pneumonia plays an important role in this group of patients [12]. The other study demonstrated that the functional dysphagia scale could be used to predict aspiration pneumonia in PD patients [13]. Furthermore, ventilatory dysfunction including lung volume, respiratory muscle weakness, and sleep breathing disorders were all affected in PD patients [14]. This would make PD patients more vulnerable to lung infections and unlikely to recover.

Bladder dysfunction is also a common nonmotor disorder in PD patients [15, 16]. Symptoms include nocturia, urgency, and frequency. PD patients had problems completely emptying their bladders, which led to bacterial growth. This could explain why PD patients were more likely to have urinary tract infections than their age- and sex-matched counterparts. However, those infections were less lethal than respiratory tract infections or the other infection sites in our study.

Our data also showed that intra-abdominal infections were less common in PD patients. PD patients were susceptible to gastrointestinal problems including gastroparesis, decreased bowel movement frequency, and anorectal dysfunction [17]. However, in contrast to bladder and ventilator dysfunction, this situation did not make PD patients more vulnerable to intra-abdominal infections.

In our study, PD patients had fewer comorbid underlying diseases than the control group, including liver cirrhosis, malignancy, congestive heart failure, and autoimmune disease. These findings are compatible with Vossius's study in which PD patients were less likely to have cardiovascular disease and cancer than non-PD patients [18]. These comorbidities would lead to higher mortality rates in the situation of sepsis and may explain the lower mortality rate in PD patients with serious infection (7.2% vs. 14.1%). However, after we created the multivariate logistic regression model, we found that PD itself still had protective effect against sepsis-related mortality with an OR of 0.616. Akbar also found that PD patients with aspiration pneumonia had lower mortality rates than non-PD patients (17% vs. 22%) [19].

We found that PD patients had less fulminant clinical courses than non-PD patients demonstrated by the inflammatory and organ dysfunction biomarkers and clinical outcomes. On the other hand, although their sepsis severity was lower, PD patients had longer hospitalizations than their counterparts. Clinical studies revealed that PD patients had higher rate of frailty [20, 21], and it could make them look sicker and therefore seek medical help earlier and could also lead to longer hospital stay [21]. The other study also showed that PD patients had a greater chance of ED admission due to pneumonia, hip fracture, and urinary tract infection as well as longer hospital stays [4]. Our study showed that PD patients with sepsis need 2–3 more days in the hospital than sepsis patients without PD. This discrepancy showed that PD patients may need more time to recover from sepsis. Studies have shown that an inflammatory process may have harmful consequences on neurodegeneration in PD [22]. And since sepsis will provoke a pathogen-induced cytokine storm and body-wide inflammation, a certain impact on PD patients could be expected [23].

In this study, the culture rate and bacterial spectra were similar between the PD and non-PD groups. Although the culture results may not strongly represent sepsis severity or predict mortality, they could provide a clearer picture of sepsis events and guide the treatment [24]. However, as pneumonia is the most important infection source of sepsis in PD patients from the view of prevalence and disease severity, the positive rate of blood culture for hospitalized pneumonia in ED is around 7%, and only half of them affected management [25]. Two-thirds of the blood cultures in PD patients were positive for gram-negative bacteria, which mainly came from the genitourinary tract and the gastrointestinal tract. Nevertheless, our data showed that, in bloodstream infections, PD patients were not different from their counterparts.

The inflammatory biomarker results demonstrated their clinical application in septic PD patients. Like in our daily practice, bandemia, serum CRP, and lactate levels could be used to diagnose sepsis or predict its severity in PD patients [26–28]. However, since PD patients have less severe sepsis than non-PD patients admitted to the ED, serum lactate levels were checked far less often in PD patients in the ED than CRP and differential white blood cell counts. The AUC of lactate for predicting 28-day sepsis-related mortality was thus lower than that of previous published data [29].

### 4.1. Limitations

Since this was a retrospective study, the clinical features including disease duration, PD severity (e.g., Unified Parkinson's Disease Rating Scale and Hoehn and Yahr staging scores), and daily dose of anti-Parkinsonian agents (equivalent dose of levodopa) could not be obtained from the computerized database, although infections often occurred in patients with advanced PD.

## 5. Conclusions

Sepsis occurred in PD patients, and respiratory tract and urinary tract infections were the two most common infectious sources. Empiric therapy should be based on experience and cover both respiratory tract and urinary tract infections. Although the clinical courses were more benign in septic patients with PD, they had longer hospitalizations than their non-PD counterparts. Early diagnosis and treatment are essential for survival.

## Figures and Tables

**Table 1:** Demographics and clinical characteristics of septic patients with or without Parkinson's disease.

Variable	Non-PD	PD	P value
	(n = 2,400)	(n = 1,200)	
Age, mean ± SD	77.3 ± 8.8	77.3 ± 8.8	0.997
Sex, male, n (%)	1313 (54.7%)	623 (51.9%)	0.119
Vital signs at ED (mean ± SD)			
Body temperature (°C)	37.3 ± 1.1	37.5 ± 1.2	<0.001^*∗*^
Heart rate (beats per minute)	99.3 ± 23.4	98.8 ± 21.9	0.484
Systolic blood pressure (mmHg)	140.4 ± 36.8	138.9 ± 34.8	0.239
Diastolic blood pressure (mmHg)	80.2 ± 29.5	79.9 ± 33.3	0.834
Respiratory rate (breaths per minute)	20.4 ± 3.9	20.4 ± 3.6	0.972
Major comorbidities, n (%)			
Liver cirrhosis	186 (7.8%)	33 (2.8%)	<0.001^*∗*^
Hypertension	1156 (48.2%)	605 (50.4)	0.203
Diabetes mellitus	863 (36.0%)	443 (36.9%)	0.581
Chronic renal insufficiency	247 (10.3%)	115 (9.6%)	0.518
Congestive heart failure	271 (11.3%)	71 (5.9%)	<0.001^*∗*^
Malignancy	547 (22.8%)	114 (9.5%)	<0.001^*∗*^
Hematologic disorder	58(2.4%)	9(0.8%)	<0.001^*∗*^
Autoimmune disease	33(1.4%)	7(0.6%)	0.041^*∗*^
Major source of infection, n (%)			
Respiratory tract	1046 (43.6%)	594 (49.5%)	0.001^*∗*^
Urinary tract	559 (23.3%)	583 (48.6%)	<0.001^*∗*^
Soft tissue infection	143 (6.0%)	87 (7.2%)	0.148
Meningitis	21 (0.9%)	5 (0.4%)	0.147
Intra-abdominal infection	87 (3.6%)	17 (1.4%)	<0.001^*∗*^
Biliary tract infection	170 (7.1%)	29 (2.4%)	<0.001^*∗*^
Others	656 (27.3%)	140 (11.7%)	<0.001^*∗*^
Bacteremia, n (%)	331 (13.8%)	161 (13.4%)	0.797
Days of admission (mean ± SD)	15.4 ± 13.5	17.8 ± 13.0	<0.001^*∗*^
Days of admission (survivors only)	15.5 ± 13.0	18.1 ± 12.9	<0.001^*∗*^
ICU admission, n (%)	306 (12.8%)	124 (10.3%)	0.038^*∗*^
Septic shock within 72 hours, n (%)	174 (7.2%)	60 (5.0%)	0.010^*∗*^
Respiratory failure within 72 hours, n (%)	365 (15.2%)	122 (10.2%)	<0.001^*∗*^
28-day in-hospital mortality, n (%)	339 (14.1%)	86 (7.2%)	<0.001^*∗*^

PD: Parkinson's disease; ED: emergency department; SD: standard deviation; ICU: intensive care unit

^*∗*^P < 0.05

**Table 2:** Presentation of laboratory data of septic patients with or without Parkinson's disease.

Variable	Non-PD	PD	P value
	(n = 2,400)	(n = 1,200)	
WBC (1000/mm^3^), mean ± SD	12.4 ± 10.5	11.9 ± 5.4	0.042^*∗*^
Segment (%), mean ± SD	78.3 ± 13.6	80.0 ± 11.4	<0.001^*∗*^
Lymphocytes (%), mean ± SD	13.0 ± 10.2	12.4 ± 8.8	0.06
Band form (%), mean ± SD	0.9 ± 2.7	0.7 ± 2.9	0.07
C-reactive protein (mg/L), mean ± SD	97.0 ± 92.2	83.6 ± 83.3	<0.001^*∗*^
Lactate (mg/dL), mean ± SD	29.8 ± 31.8	25.0 ± 25.2	0.024^*∗*^
BUN (mg/dL), mean ± SD	31.5 ± 28.8	26.6 ± 20.9	<0.001^*∗*^
Creatinine (mg/dL), mean ± SD	1.8 ± 1.9	1.3 ± 1.0	<0.001^*∗*^
Bilirubin (mg/dL), mean ± SD	2.7 ± 4.2	1.3 ± 1.3	<0.001^*∗*^
GOT (U/L), mean ± SD	107.3 ± 503.6	60.0 ± 189.8	0.009^*∗*^
Sodium (mEq/L), mean ± SD	134.8 ± 7.0	134.5 ± 8.0	0.259
Potassium (mEq/L), mean ± SD	4.0 ± 0.9	4.0 ± 0.7	0.227

PD: Parkinson's disease; WBC: white blood cells; SD: standard deviation; BUN: blood urea nitrogen; GOT: glutamic oxaloacetic transaminase

Normal range of WBC ( 3.5~11), Segment (42-74), Lymphocyte (20-56), Band form (0-3), C-reactive protein (<5), Lactate (4.5-19.8), BUN (6-21), Creatinine (0.44-1.27), Bilirubin (0.2-1.4), GOT (0-37), Sodium (134-148), Potassium (3.5-5.2)

^*∗*^P < 0.05

**Table 3:** Blood culture results for septic patients with and without Parkinson's disease.

Bacterial strain	Non-PD	PD	P value
	(n = 2,400)	(n = 1,200)	
Gram-negative	208 (8.7%)	103 (8.6%)	0.215
*Escherichia coli*	115 (4.8%)	60 (5.0%)	
*Klebsiella pneumoniae*	39 (1.6%)	15 (1.3%)	
*Proteus mirabilis *	5 (0.2%)	12 (1.0%)	
*Pseudomonas aeruginosa*	8 (0.3%)	3 (0.3%)	
*Salmonella enterica*	5 (0.2%)	1 (0.1%)	
Other	5 (0.2%)	12 (1.0%)	
Gram-positive	111 (4.6%)	57 (4.8%)	
*Staphylococcus aureus*	35 (1.5%)	14 (1.2%)	
*Staphylococcus spp.*	32 (1.3%)	12 (1.0%)	
*Streptococcus spp.*	34 (1.4%)	27 (2.3%)	
*Streptococcus pneumonia*	3 (0.1%)	2 (0.2%)	
Other	8 (0.3%)	9 (0.8%)	
Anaerobic bacterium	11 (0.5%)	0 (0%)	
Fungus	1 (0.0%)	1 (0.1%)	

PD: Parkinson's disease

**Table 4:** Comparison of survivors and nonsurvivors of septic Parkinson's disease within 28 days of admission.

Variable	Survivors	Nonsurvivors	P value
	(n = 1,114)	(n = 86)	
Age (years), mean ± SD	77.1 ± 8.9	79.6 ± 7.0	0.011^*∗*^
Sex, male, n (%)	589 (52.9%)	34 (39.5%)	0.017^*∗*^
Vital signs at ED, mean ± SD			
Body temperature (°C), mean ± SD	37.5 ± 1.2	37.3 ± 1.4	0.167
Heart rate (beats per minute), mean ± SD	98.5 ± 20.9	102.9 ± 32.1	0.216
Systolic blood pressure (mmHg), mean ± SD	139.9 ± 34.0	125.0 ± 41.8	0.002^*∗*^
Diastolic blood pressure (mmHg), mean ± SD	80.6 ± 33.8	71.0 ± 24.4	0.013^*∗*^
Respiratory rate (breaths per minute), mean ± SD	20.3 ± 3.3	20.7 ± 6.1	0.653
Major comorbidities, n (%)			
Liver cirrhosis	28 (2.5%)	5 (5.8%)	0.081
Hypertension	568 (51.0%)	37 (43.0%)	0.155
Diabetes mellitus	421 (37.8%)	17 (25.6%)	0.024^*∗*^
Renal insufficiency	96 (8.6%)	19 (22.1%)	<0.001^*∗*^
Congestive heart failure	61 (5.5%)	10 (11.6%)	0.020^*∗*^
Malignancy	98 (8.8%)	16 (18.6%)	0.003^*∗*^
Hematologic disorder	7 (0.6%)	2 (3.0%)	0.086
Autoimmune disease	7 (0.6%)	0 (0%)	1.000
Major source of infection, n (%)			
Respiratory tract	531 (47.7%)	63 (73.3%)	<0.001^*∗*^
Urinary tract	563 (50.5%)	20 (23.3%)	<0.001^*∗*^
Soft tissue infection	82 (7.4%)	5 (5.8%)	0.828
Meningitis	4 (0.4%)	1 (1.2%)	0.265
Intra-abdomen infection	17 (1.5%)	0 (0%)	0.627
Biliary tract infection	27 (2.4%)	2 (2.3%)	1.000
Other	126 (11.3%)	14 (16.3%)	0.167
Bacteremia, n (%)	146 (13.1%)	15 (17.4%)	0.256
ICU admission, n (%)	100 (9.0%)	24 (27.9%)	<0.001^*∗*^
Septic shock within 72 hours, n (%)	41 (3.7%)	19 (22.1%)	<0.001^*∗*^
Respiratory failure within 72 hours, n (%)	94 (8.4%)	28 (32.6%)	<0.001^*∗*^

SD: standard deviation; ED: emergency department; ICU: intensive care unit

^*∗*^P < 0.05

**Table 5:** Presentation of inflammatory and organ dysfunction biomarkers in survivors and nonsurvivors of septic PD patients within 28 days of admission.

Biomarkers	Case numbers	Survivors	Nonsurvivors	P value
WBC (1000/mm^3^), mean ± SD	1,105/86	11.9 ± 5.3	11.9 ± 6.4	0.976
Segment (%), mean ± SD	1,103/86	80.2 ± 10.8	77.4 ± 17.6	0.031^*∗*^
Lymphocyte (%), mean ± SD	1,103/86	12.4 ± 8.4	12.3 ± 12.9	0.959
Band form (%), mean ± SD	1,103/86	0.6 ± 2.1	3.1 ± 7.2	0.002^*∗*^
C-reactive protein (mg/dL), mean ± SD	914/71	79.2 ± 78.1	139.4 ± 120.5	<0.001^*∗*^
Lactate (mg/dL), mean ± SD	220/38	21.9 ± 14.9	43.2 ± 52.0	0.016^*∗*^
BUN (mg/dL), mean ± SD	756/57	25.6 ± 20.0	39.8 ± 26.7	<0.001^*∗*^
Creatinine (mg/dL), mean ± SD	1,079/81	1.3 ± 1.1	1.5 ± 1.0	0.033^*∗*^
Bilirubin (mg/dL), mean ± SD	182/16	1.2 ± 1.2	1.9 ± 2.0	0.175

PD: Parkinson's disease; WBC: white blood cells; SD: standard deviation; BUN: blood urea nitrogen

^*∗*^P < 0.05

## Data Availability

The data used to support the findings of this study are available from the corresponding author upon request.
